# Community Healthcare Workers’ Perception of an Educational Intervention in the Care of Patients with Sickle Cell Disease in Brazil

**DOI:** 10.4084/MJHID.2015.031

**Published:** 2015-04-20

**Authors:** Ludmila Mourão Xavier Gomes, Thiago Luis de Andrade Barbosa, Elen Débora Souza Vieira, Lara Jhulian Tolentino Vieira, Karla Patrícia Ataíde Nery Castro, Igor Alcântara Pereira, Antônio Prates Caldeira, Heloísa de Carvalho Torres, Marcos Borato Viana

**Affiliations:** 1 Núcleo de Ações e Pesquisa em Apoio Diagnóstico, Federal University of Minas Gerais, Belo Horizonte, Brazil; 2 Department of Medicine, State University of Montes Claros, Montes Claros, Brazil; 3 Department of Applied Nursing, Federal University of Minas Gerais, Belo Horizonte, Brazil

## Abstract

**Introduction:**

Despite advances in the management of sickle cell disease, gaps still exist in the training of primary healthcare professionals for monitoring patients with the disease.

**Objective:**

To assess the perception of community healthcare workers about the care and monitoring of patients with sickle cell disease after an educational intervention.

**Method:**

This exploratory, descriptive, and the qualitative study was conducted in Montes Claros, state of Minas Gerais, Brazil. The intervention involved the educational training of community healthcare workers from the Family Health Program of the Brazilian Unified Health System. The focus group technique was used to collect the data. The following topics were covered in the discussion: assessment of educational workshops, changes observed in the perception of professionals after training, profile of home visits, and access to and provision of basic healthcare services to individuals with sickle cell disease. The discussions were tape-recorded and transcribed verbatim. The data were subjected to content analysis and empirically organized into two categories.

**Results:**

Changes in the healthcare practices of community health workers were observed after the educational intervention. The prioritization of healthcare services for patients with sickle cell disease and monitoring of clinical warning signs in healthcare units were observed. Furthermore, changes were observed in the profile of home visits to patients, which were performed using a script provided in the educational intervention.

**Conclusion:**

The educational intervention significantly changed the work process of community health workers concerning patient monitoring in primary healthcare.

## Introduction

Sickle cell disease (SCD) is the most important hemoglobinopathy worldwide and is associated with high morbidity and mortality.^1^ In Brazil, SCD is a relevant public health topic, considering the epidemiological, clinical, economic, and social issues involved. The prevalence of sickle cell trait in Brazil is estimated to be between 2% and 8%,^2^ depending on the ethnic composition of the regional populations. The incidence of SCD in Minas Gerais, a Southeastern state, is 1:1,400 newborns, according to the Newborn Screening Program (NSP-MG).^3^

The results of the NSP-MG have been rewarding, but many challenges remain regarding patient care, including the training of healthcare professionals in educational activities and the provision of continuous patient care to reduce morbidity and mortality.^3^,^4^ Research conducted in Minas Gerais showed poor knowledge of primary care professionals about many aspects of SCD. Poor knowledge probably reflects badly on the quality of healthcare provided to people with SCD and their families.^5^

One study that evaluated the perception of patients with SCD identified several limitations related to providing primary healthcare to them. These limitations included restricted access to healthcare services, lack of communication between primary and secondary healthcare professionals, and lack of confidence in the ability of primary care professionals to accurately provide information related to the disease.^6^

Experiences of educational interventions for primary healthcare professionals about SCD are scanty. Additionally, they had limited goals such as communication of newborn screening results^7^ and communication skills to offer prenatal screening for SCD and thalassemia.^8^ There are no studies of educational interventions targeting primary healthcare professionals to address general care to patients with SCD.

In Brazil, the Family Health Program has been implemented to strengthen primary healthcare and reorganize the assistance model in the Brazilian Health System (Sistema Único de Saúde - SUS). Considering its principles and the organization of its work processes, the Family Health Program provides conditions to mitigate the indicators of suffering caused by chronic diseases such as SCD. Moreover, the Program is characterized by the integrated work of a multidisciplinary team comprising doctors, nurses, nurse technicians, and community health workers.^9^,^10^ Community health workers are essential for the Family Health Program because they reside in the same area where they work, conduct home visits, and so they can fully understand the community’s health problems, way of life, and culture.^11^ Therefore, these workers require clear and objective information from the technical–scientific area to guide community-based activities,^12^,^13^ and training of these professionals is crucial.

The aim of this study was to analyze the perception of community primary healthcare workers about the care and monitoring of individuals with SCD after an educational intervention.

## Methods

This exploratory, descriptive, and qualitative study was conducted in basic healthcare units of Montes Claros, state of Minas Gerais, Brazil. Montes Claros is situated in a region with a high prevalence of SCD. The study was conducted after the educational intervention for community health workers.

### Educational intervention

The intervention consisted of the training of community health agents in the Family Health Program on the primary care and monitoring of individuals with SCD. It was conducted by three nurses who were previously trained via a 90-hour distance learning course over a 3-month period. Activities were conducted in the form of active methodologies such as case studies, meetings, stage plays, and parodies.

The contents and design of the program are depicted in Table 1.

The educational intervention lasted 40 hours: 30 hours in the classroom and 10 hours outside the classroom. Six 5-hour meetings were conducted with healthcare professionals at intervals of 7 days. After each workshop, the professionals were assigned homework related to the topics, which would be addressed in the subsequent workshop.

On completion of training, two visits were made to healthcare units and involved discussions on the main topics and guidance on patient monitoring.

A total of 68 community health agents who assisted patients with SCD and were from six basic healthcare units were trained. Medical records from the NSP-MG were used. In addition, each healthcare team was interrogated about the presence of individuals with SCD in each jurisdiction.

### Participants

Three months after the intervention, the trained community health agents were invited to participate in a meeting in a room provided by the Health Department of Montes Claros. The professionals were selected on the basis of the following criteria: (i) worked in the Family Health Program during the study period, i.e. they were not on vacation or work leave; (ii) their jurisdiction assisted patients with SCD; (iii) they passed the course with a minimum attendance of 80%; and (iv) they agreed to participate in the study. Among the 68 trained community health agents, 27 worked in jurisdictions containing patients with SCD, and two refused to participate in the study. Consequently, 25 community health workers were selected to participate in this study.

### Data collection

The focus group technique was used, allowing interaction and discussions of aspects related to training and changes implemented in daily activities involving patient care and monitoring.^14^ For the focus discussion, a moderator and two observers who had not participated in the educational intervention were present. The focus groups sessions lasted no more than a hundred minutes and used a plan that included the following topics: assessment of educational workshops, changes observed in the professionals after training, profile of home visits, access to and provision of basic healthcare to individuals with SCD. The discussions were tape recorded and later transcribed verbatim. The focus sessions were conducted with two groups, one with 12 participants and the other with 13. The professionals were allocated to each focus group on the basis of their availability on previously scheduled dates. Each group participated in only one focus session. The number of groups formed for the focus sessions allowed the data to reach saturation range, ensuring that no new or relevant data were missing when data collection was completed.

### Analysis of the data

The data were subjected to thematic content analysis according to the following steps: preanalysis, content analysis, processing of the results, and interpretation.^15^ Subsequently, the data were organized into two empirical categories. Statements from participants were identified by letter codes accompanied by Arabic numerals. The two focus groups were designated G1 and G2, and participants received a code with the letter P.

### Ethical aspects

The confidentiality and anonymity of study participants were guaranteed throughout the study. This study was approved by the Research Ethics Committee and was registered in the Brazilian National Council of Research Ethics under protocol CAAE-0683.0.203.000-11.

## Results

The profile of the 25 professionals is presented in Table 2.

In the focus group, two categories emerged based on the participants’ statements, as follows: “perception of the educational intervention” and “training for the promotion of changes in the work of the professionals.”

### Perception of educational intervention

In this category, the professionals discussed the topics and evaluated training performed. The educational intervention received a positive assessment from participants, and they gained deeper knowledge about different aspects of patient care and monitoring:

“Training was great, since I had the freedom to play, role play, relax, and clarify many topics. On our own, we were looking for knowledge about the disease through the problems we encounter in our daily lives.” G2P4

“We didn't know about priapism, wounds, the natural history of the disease, the age of each medication, electrophoresis, and enlarged spleen.” G2P10 and G2P8

Community health workers covered many topics related to SCD that they were unaware of; these topics were addressed in workshops. Negative experiences prior to training were mentioned by the professionals. Among these, the most important was the lack of awareness about priapism:

“I had a very bad negative experience, I went to the house of a young man with SCD who had an erection at the time of the visit. I left in a hurry because I didn't know what priapism was. Today I know how to handle this situation.” G2P10

It was noted that before the intervention, some professionals stated that they were unaware on how to manage patients with SCD in healthcare units. The course taught them how to monitor patients, and the professionals considered the educational intervention to be relevant to their everyday practice:

“This course was very important to our practice. By knowing all these aspects, now we can take better care of the person who has this disease. Now we pay more attention when a person with SCD arrives in our unit.” G1P1 and G1P7.

“The course made me think about my own work. I didn't pay much attention to my patients SCD, but the course awakened my interest in caring.” GP1P2

### Training to promote changes in the routine work of professionals

Of note, this category included changes implemented by community health agents in their practice after training (Table 3). They discussed changes in areas such as the prioritization of services, treatment of new patients, home visit routines, and monitoring of specific outcomes of patients with SCD. These changes are described below as subunits of this thematic category.

### Subtopic: Prioritizing care and treating new patients in the health unit

Prior to training, healthcare professionals lacked knowledge regarding the need for prioritizing the care for patients with SCD. They were not able to understand why mothers of children with the disease were so critical regarding delays in care for their children and kept insisting on it to be prioritized:

“It was in the course that I understood the issue of priority of care. I started thinking about the mother in my area who has two boys with SCD, and who comes to the unit and requests her children to be assisted quickly, unlike other mothers of children who do not have SCD.” G1P10

After training, the professionals stated that they prioritized care for the child visiting their unit with warning signs. Furthermore, they promptly provided humanized care services to individuals with SCD.

“When the mother comes in, I inquire whether the child has fever, because it is a risk situation. I also note if the child has pain in the belly, which can be a problem in the spleen.” GP2P12, G2P8.

Disease surveillance was performed for every child who visited the healthcare unit to have anthropometric measurements. In cases where the child had any health problems or warning signs, health services were performed promptly:

“The child came to get weighed and was complaining of fever. So I went and talked to the doctor. Then the child was examined promptly and the problem was solved.” GP2P4

### Subtopic: changes in the home visit routine

The main changes referred to by the professionals occurred in home visits, which were performed using systematized scripts introduced in the course. Community health agents reported that home visits to patients with SCD became more time consuming because these professionals were concerned about evaluating other health aspects and providing targeted orientation. In addition, the visits became prioritized, i.e., the professionals made their monthly visits starting with families with the greatest risk, which included the homes of patients with SCD:

“The visit takes much longer. I see if the child in my area is taking folic acid and antibiotics to prevent complications and remember of the vaccination programs. I teach them about palpating the spleen and warning signs. I didn't do this prior to the course.” G1P11

According to the professionals, the primary change in home visits after training was monitoring the use of folic acid, prophylactic antibiotics, and vaccines in patients as well as teaching them how to palpate the spleen and diagnose warning signs.

In home visits, orientations on precautions about environmental conditions were conducted, and the need for constant hydration was also taught, improving care for her child:

“In one case, the mother worked by selling door-to-door and used to take the child with her, exposing the child to the sun, which in turn led to bouts of pain. So I told the mother about the risk of dehydration, and now she is avoiding taking the child with her, and always hydrates her, and this is the result of the course. G1P9

## Discussion

The educational intervention received a positive evaluation from community health agents. In addition, the intervention produced changes in the daily work of these professionals.

Because of its dialectic and problem-based approach as well as the use of various educational resources that allowed participants to interact, the educational intervention increased awareness of community health agents about the care and monitoring of individuals with SCD. During training workshops, the professionals reflected on their role in patient care. The dialectic approach of educational activities provided individuals with the opportunity of self-reflection and to consider themselves as promoters of change. In this sense, the educational intervention aimed to contribute to the pursuit of transformation, and changes were produced following training.^16^

The experiences and problems described by healthcare workers during professional life in the health team redirected their attention to training with the purpose of improving care. In this context, continuing education is considered important and is understood as “learning at work, in which learning and teaching are incorporated into the daily lives of organizations and work”.^17^ The aim of continuing education is the training of health workers, considering their own experiences and the problems they face.

In the perception of these professionals, the educational intervention led to changes in their healthcare practices. One of these changes was the prioritization of care for patients with SCD showing warning signs of acute events, such as fever, pain, sudden increase in pallor, worsening jaundice, abdominal distension, enlarged spleen or liver, cough or difficulty in breathing, priapism, neurological changes, inability to swallow liquids, dehydration, vomiting, and hematuria.

The professionals’ statements indicated that mothers of children with SCD were more demanding and required immediate care for their children. This fact can be explained by the occurrence of vaso-occlusive phenomena that can lead to serious complications such as stroke when a child showing warning signs is not treated quickly. Knowledge derived from the educational intervention allowed the provision of better care because it changed the professionals’ perception of the mothers who demand immediate care for their children during emergencies. In their daily work, community health agents alerted other team members to expedite care as soon as they detected warning signs.

A relevant point in the professionals’ statements was related to patient monitoring. The professionals were aware of the warning signs and always interrogated the responsible parties about these signs. This happened, for example, when the child visited to the clinic for anthropometric measurements and any complaints were noted by the professionals or reported by the mother or guardian. A routine appointment can then become an urgent situation for community health workers.

Surveillance activities undertaken by community agents are not restricted to the health units. A study of primary healthcare professionals in Brazil found that community workers have acted more intensely in the areas of education and coordination of information between the healthcare team and service users. Moreover, they performed various outdoor activities in streets, homes, and reference points in the community. Home visits were the main activity of these professionals.^18^ Another study found that home visit procedures conducted by community health workers were not standardized and were defined by each professional. Moreover, home visits are scheduled on the basis of their experience and physical location within each service region. Home visits, therefore, become bureaucratic reproductions of medical consultations, where forms are completed and routine updates are provided, limiting the establishment of a relationship between the healthcare team and service users.^19^

In patients with SCD, patient monitoring through regular, targeted home visits is essential to achieve more effective therapeutic results. The results of the present study showed that home visits became systematized and targeted to patients with SCD after adoption of a script provided in the educational intervention. Moreover, changes were observed in the prioritization of home visits to patients with SCD because these families are at a greater risk. In this respect, the prioritization of home visits benefited not only individuals with SCD but also families that constituted risk groups. In the systematized home visits, the professionals guided patients on the use of folic acid, prophylactic antibiotics, and vaccines and taught family members how to palpate the child’s spleen. Monitoring the use of folic acid in home visits is necessary to cope with accelerated erythropoiesis, a specific feature of the disease.^20^ Adherence to drug therapy is a relevant issue in patient monitoring, as previously reported.^21^,^22^ When community health agents noted poor adherence to the recommended drug therapy in home visits, these visits became more time consuming because of the constant need to explain the importance of preventive healthcare measures to families.

The vaccination status should be monitored during visits both in relation to the basic vaccination schedules and the vaccines recommended for SCD. Previous studies indicated that vaccination coverage in relation to the basic calendar varied between 65% and 100%.^4^,^23^ However, this is not the reality for all vaccines. In Brazil, vaccines for *pneumococcus* and *meningococcus* were included in the basic immunization schedule in 2010. Prior to this date, two studies in the Brazilian states of Minas Gerais and Espírito Santo showed that 43.8% and 50% of children with SCD, respectively, had incomplete immunization against *pneumococcus*.^4^,^24^ A study in London found that immunization against encapsulated bacteria and the flu virus was also precarious among adults and children with SCD.^25^ In Madrid, the regular vaccine coverage was 85%. It was 50% for flu virus and 15% for hepatitis A.^26^

Home visits are also conducive to teaching parents how to palpate the spleen for the early diagnosis of acute splenic sequestration. Instructing parents on this technique can decrease mortality, considering that this outcome can quickly lead to death. Other educational activities conducted by these professionals also cover environmental education and the perception of warning signs. Families are instructed to keep patients hydrated and away from adverse environmental conditions, such as extreme heat and cold. The diagnosis of warning signs allows parents to identify changes that demand emergency care considering the level of complexity required. This information may help reduce patient mortality and prevent sickle cell crises.

The limitations of the present study are related to data collection. The focus group has limited ability to make inferences about large population groups and cannot test hypotheses in experiments.^14^ Another limitation derives from the lack of interviews with the same participants to compare individualized results of the focus discussions. In this respect, participants may change their opinions during the focus discussions because of the positioning of the researchers about sensitive issues. In addition, the effects of training community agents on patients’ health were not assessed because these effects were not included in the study objectives.

## Conclusions

The educational intervention on SCD produced changes in the attitudes of healthcare professionals to improve the quality of care provided to individuals with SCD. Knowledge acquired in training changed attitudes and improved their skills.

Based on these results, we suggest the widespread training of community health workers who treat SCD patients in their service area. The educational intervention studies that include higher-level primary healthcare professionals are also recommended.

Considering the limited knowledge about SCD and primary healthcare, further studies are recommended, particularly those that directly assess the effects of training on the health of patients with SCD.

## Figures and Tables

**Table 1:** Issues discussed in educational workshops held with the community health agents

Workshop 1	Presentation of the course Agreement on the objectives of the course Pre-test application Introduction to sickle cell disease (SCD) and differences between SCD and sickle cell trait Origin, epidemiology, pathophysiology, and clinical manifestations of SCD Importance of monitoring the disease in the primary care set Practical task: create a play or parody about painful crisis or priapism in a group of 4 or 5 people
Workshop 2	Theater or parody presentation prepared by the groups Health surveillance activities for children with SCD in a basic health unit Growth and development of children with SCD Use of folic acid and prophylactic antibiotics Practice of spleen palpation Warning signs of acute events in SCD Prioritization of service Systematization of home visits Practical task: doing home visits using a script available in the flipchart and discussed during the workshop
Workshop 3	Presentation and discussion of home visits to the families of children with SCD Discussion of the main issues about the health of adolescents with SCD (growth and development, vaccines, use of folic acid, priapism, leg ulcers) Family planning Aspects involving adult health with SCD Living problems of patients with SCD Task: studying and preparing oral presentations of specific issues (pregnancy, nutrition, school life, physical activity, oral health) in groups
Workshop 4	Pregnancy Nutrition School life Physical activity Oral health Monitoring of families Use of family heredograms and eco-maps Task: creating a heredogram and an eco-map of a family with SCD
Workshop 5	Discussion of the heredogram and family eco-map with SCD Rights and duties of the person with SCD Other diseases detected by the Newborn Screening Program in Minas Gerais Task: “How does the Call Center for Hemoglobinopathies work?”
Workshop 6	Institutions assisting people with SCD The important role of the Call Center for Hemoglobinopathies Implementation of changes on the care of people with SCD Evaluation of the educational intervention Post-test application

**Table 2:** Demographic characteristics of participants in the study

Variable	N	%
Gender		
Female	20	80
Male	05	20
Age		
20 to 29.9 years	13	52
30 to 39.9 years	10	40
40 to 50 years	2	8
Marital status		
Married or stable relationship	14	56
Single	07	28
Divorced	01	04
Widow	03	12
Number of children		
None	09	03
1–2	12	48
3 or more	04	16
Length of Service in Primary Care		
<1 year	09	36
1 to 5 years	03	12
5.1 to 9 years	09	36
≥10 years	04	16

**Table 3:** Routine work of professionals before and after intervention

Routine work of professionals before intervention	Changes in the routine work of professionals after intervention
Care for patients with sickle cell disease was not prioritized for those with warning signs of acute clinical events	Care for the child with warning signs of acute events was prioritized
Professionals were not conducting home visits in a systematized way	Systematized scripts for home visits suggested during the course have been used since then
Home visits to patients with SCD were not prioritized	Home visits have become prioritized by professionals. The monthly visits now start to families with the greatest risk, which included the homes of patients with SCD.
Home visits did not monitor the use of folic acid, prophylactic antibiotics, and specific vaccines to people with sickle cell disease. Education about warning signs of acute events and teaching of abdominal palpation to help families in the diagnosis of acute splenic sequestration were never done	In home visits the professionals started monitoring the use of folic acid, prophylactic antibiotics, and vaccines. The technique of spleen was taught to the families and warning signs of acute events were discussed with them
Environmental education (warming in cold weather and refreshing in hot environment) and constant hydration were not recognized as important preventive actions for patients with SCD	In home visits, constant hydration was enphasized. Precautions about environmental conditions were discussed with the patient and his/her family
